# An optimized nucleic acid isolation protocol for virus diagnostics in cassava (*Manihot esculenta* Crantz.)

**DOI:** 10.1016/j.mex.2021.101496

**Published:** 2021-08-21

**Authors:** Jenyfer Jimenez, Ana Maria Leiva, Cristian Olaya, Daniela Acosta-Trujillo, Wilmer Jose Cuellar

**Affiliations:** Virology Laboratory, Crops for Nutrition and Health, International Center for Tropical Agriculture (CIAT), AA 6713, Cali, Colombia

**Keywords:** CTAB extraction, Silica gel, Disease surveillance, Begomovirus, Potexvirus

## Abstract

Our group works on the detection and characterization of cassava viruses, supporting projects that involve large scale pathogen surveillance activities and resistance screening assays in multiple and remote locations. In order to comply with these applications, nucleic acid isolation protocols need to be cost effective, adjusted for samples that will stand long distance transport and harsh storage conditions, while maximizing the yield and quality of the nucleic acid extracts obtained. The method we describe here has been widely used and validated using different downstream tests (including, but not limited to, Rolling Circle Amplification and Illumina and Nanopore sequencing), but is currently unpublished. The protocol begins with milligram amounts of dry leaf samples stored in silica gel, does not require liquid Nitrogen nor phenol extraction and produces an average of 2.11 µg of nucleic acids per mg of dry tissue.

• DNA purity estimations reveal OD260/280 ratios above 2.0 and OD260/230 ratios above 1.7, even for samples stored in silica gel for several months.
• The high quality of the extracts is suitable for detection of DNA and RNA viruses, with high efficiency.
• We suggest this method could be used as part of a gold standard kit for virus detection in cassava.

## Graphical abstract

**Figure:**
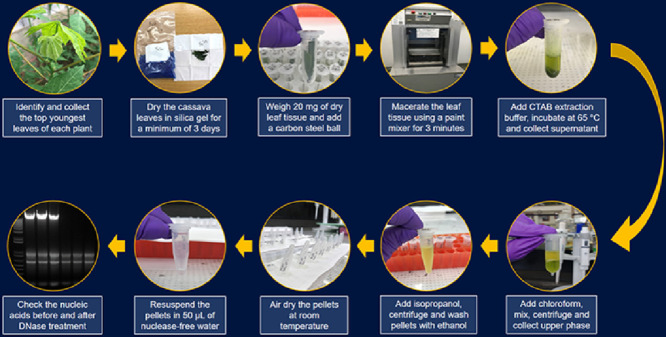

Specifications table

**Table:** 

Subject Area:	*Agricultural and Biological Sciences*
More specific subject area:	*Molecular Biology*
Method name:	*Nucleic acid extraction protocol for the detection and characterization of cassava-infecting viruses*
Name and reference of original method:	*Doyle, J. (1991). DNA protocols for plants. In Molecular techniques in taxonomy (pp. 283-293). Springer, Berlin, Heidelberg.*
Resource availability:	*n/a*

## Materials and methods

### Reagents and consumables

• Silica gel blue (Profinas S.A., Colombia)
• Cetyltrimethylammonium bromide (CTAB; Merck)
• Tris-hydrochloride (Tris–HCl; Sigma)
• Disodium ethylenediamine (EDTA; Sigma)
• Polyvinylpyrrolidone (PVP-10; Sigma)
• 2-Mercaptoethanol (βME; Merck-Millipore)
• DNase I (Promega)
• GelRed® nucleic acid gel stain 10,000X (Biotium, USA)
• 1 KB Plus DNA Ladder (Invitrogen, USA)
• Ethanol absolute (Merck-Millipore)
• Isopropanol (Merck-Millipore)
• Chloroform (Merck)
• 2X PCR Master Mix (GoTaq® Green Master Mix, Promega, USA)
• Gel loading buffer BlueJuice™ 10X (Invitrogen, Lithuania)
• 1.5 and 2.0 ml Seal-Rite® microfuge tubes (USA Scientific)
• Carbon steel balls 1.8”
• Nuclease-free tips (Tip One, USA Scientific)
• RQ1 RNase-Free DNase I (Promega, USA)

### Solutions

• CTAB Extraction buffer 1X (100 mL); modified from Doyle al. [7]: 2% (w/v) PVP, 2% (w/v) CTAB, 100 mM Tris−HCl (pH 8.0), 2 M NaCl and 25 mM EDTA (pH 8.0). Add 200 µL of 2-Mercaptoethanol before use.
• TAE 1X buffer: 1 mM EDTA (pH 8.0) and 10 mM Tris−HCl (pH 8.0).

### Equipment

• Paint mixer model FM HARBIL 5G-HD (The Cary Company, USA)
• Micropipettes (Eppendorf, P-20, P-200 and P-1000)
• Centrifuge AccuSpin Micro 17R (Fisher Scientific)
• Water bath (Thermo-Scientific)
• Thermal cycler Mastercycler nexus® (Eppendorf)
• NanoDrop 2000 (Thermo Scientific™)
• Gel documentation system Gel-Doc XR+ imager (Bio-Rad)
• Agarose gel electrophoresis chamber (BIO-RAD)
• Laboratory Analytical Balance (Mettler Toledo USA)

### Sample collection

As a standard procedure, we collect 400–600 mg of the top youngest leaves (sprouts) of each cassava plant. Wrap the leaves in tissue paper (Kimwipes®) and place them in 10 × 10 cm Ziploc® bags containing 50 g of silica gel. Silica gel is a low-cost common use desiccant that stays in solid state when saturated with water. We used a silica gel that has a blue color indicator, which turns pink when hydrated, and can be reused several times after dehydration using an oven. After collecting 4 paper pockets per bag, gently remove the air before sealing and allow the samples to dry inside the bags for at least 3 days; silica gel is replaced if it turns pink before the drying period is completed. The samples are processed as soon as they arrive to the laboratory, they can be stored at room temperature for a couple of weeks or in a cold room at 4 °C, for long-term storage.

### Total nucleic acid isolation

This protocol is adapted from that described by Doyle [7], and is used in our laboratory as a routine protocol for screening large numbers of samples. Depending on the equipment availability, one person can readily process 48 samples per day.

1. Transfer 20 mg of dried leaf tissue to a 2 mL microfuge tube containing 1 carbon steel ball and grind the samples by shaking for 3 min using a paint mixer at room temperature (RT).
2. Carefully open the tubes, add 1 mL of CTAB extraction buffer, shake vigorously using a vortex until you get an homogeneous suspension and centrifuge the tubes for 3 min at 13.000 RPM at RT.
3. -Collect 750 µL of the extract without disturbing the pellet and transfer it to a new 2 mL microfuge tube. Incubate the samples at 65 °C in a water bath for 15 min and place the tubes on ice.
4. Add one volume (750 µL) of ice-cold chloroform, mix for 1 min using a vortex until you observe a continuous phase, and centrifuge the tubes at 13.000 RPM for 10 min at 4 °C.
5. Transfer the supernatant to a new 1.5 mL microfuge tube and add one volume of ice-cold Isopropanol. Mix gently by inverting the tubes several times and incubate the tubes at -20 °C for at least 30 min.

*You can stop the protocol at this step.*

6. Centrifuge the tubes at 13,000 RPM for 15 min at 4 °C and then quickly discard as much of the supernatant by inverting the tube once, making sure the pellet stays at the bottom of the tube.
7. Wash the pellet with 500 µL of 70% ice-cold ethanol and centrifuge a 13,000 RPM for 5 min. Discard the ethanol by inverting the tube once and air dry the pellets on the bench, for at least 15 min.
8. Resuspend the pellet in 50 µL of nuclease free water and proceed to check the quality and concentration of the nucleic acid extracts by using a Nanodrop®, followed by agarose gel electrophoresis, as described below.

**Note**: Absorbance ratios OD260/280 above 1.8 were considered as indicators of a good quality isolation. For RNA virus detection proceed with DNase I treatment.

### Agarose gel electrophoresis

Resolve 4 µL of the extracts in 1% agarose gel electrophoresis using 1X TAE as running buffer. To visualize the nucleic acids use 2 µL of GelRed 10,000X per 100 mL of agarose gel volume. Photographs are taken using a Gel-Doc imager.

### DNase I treatment

For DNA virus detection, it was not necessary to treat the samples with RNase A before PCR, nevertheless for RNA virus detection we recommend treating the samples with DNase I before cDNA synthesis. For this add 5 µL of DNase 10X buffer and 1 µL of DNase I to the 50 µl of total nucleic acid elution and incubate at 37 °C for 1 h. Stop the digestion with 1 µL de DNase stop solution, incubate at 65 °C for 5 min and then transfer the tubes on ice.

### Detection of DNA and RNA viruses in infected samples

A set of confirmed virus infected samples from Southeast Asia (SEA) and South America (SA) were used to test the efficiency of the protocol to detect DNA and RNA viruses in leaf samples that were stored in silica gel for several months. A total of 115 and 44 dried leaf samples from confirmed infected plants were used for virus diagnostics by PCR and RT-PCR, respectively.

**PCR**: Sri Lankan cassava mosaic virus (SLCMV; Fam. *Geminiviridae*) is an emergent pathogen in SEA [18]. It is a circular single stranded DNA virus and one out of 11 geminiviruses that can affect cassava production worldwide [10]. For detection of SLCMV we diluted total nucleic acid extracts to a working concentration of 60 ng/µL and used 1µL of this for PCR. The total reaction volume was 20 µL using a 2X PCR Master Mix and 10 µM of each primer, SLCMV-F (5′-ATGTCGAAGCGACCAGCAGATATAAT-3′) and SLCMV-R (5′-TTAATTGCTGACCGAATCGTAGAAG -3′) . These primers amplify a region of 771 bp corresponding to the AV1 gene (Coat Protein) and were previously validated to specifically detect SLCMV from field-collected cassava samples in Thailand [18]. The PCR program, carried out in a Mastercycler Nexus® was as follows: initial denaturation at 94 °C for 5 min followed by 35 cycles of denaturation at 94 °C x 30 s, annealing at 58 °C x 30 s and extension at 72 °C x 1 min. After a final extension at 72 °C x 5 min PCR products were resolved by agarose gel electrophoresis as described above.

**RT-PCR**: Cassava common mosaic virus (CsCMV; Fam. *Alphaflexiviridae*) is a re-emergent RNA virus infecting cassava in the Americas [4,6,8]. We have collaborated with research groups in Peru, Argentina and Colombia to detect and characterize the virus using the described protocol [13,20]. Three µg of RNA were used for cDNA synthesis using 200 ng of random hexamer primers (Thermo Fisher Scientific, USA) plus 1 µL of M-MLV Reverse Transcriptase in 20 µL total reaction volume. For PCR we use 2 µL of cDNA reaction mix and primers CsCMV-3269-F (5′-GAGGCTCTTCTCTGGGAAAC-3′) and CsCMV-3896-R (5′-CTTGAGTCCAGTTTGATGTC-3′) [11,13]. These primers amplify 647 bp of the conserved RNA-dependent RNA polymerase (RdRp) domain [11,13]. The PCR program was as follows: 95 °C x 2 min followed by 35 cycles of 94 °C x 30 s, annealing at 56 °C x 30 s and extension at 72 °C x 1 min. After a final extension at 72 °C x 5 min PCR products were analyzed by agarose gel electrophoresis.

## Results and discussion

**Fig. 1:**
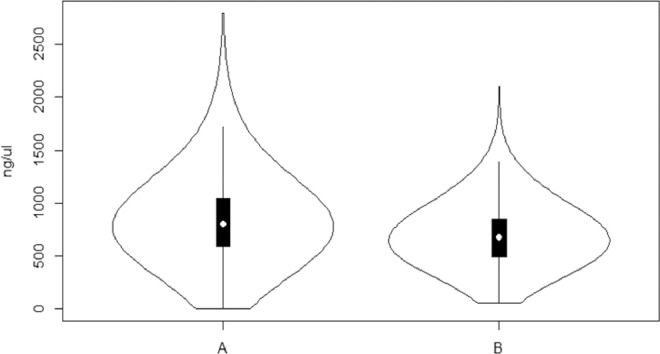
Distribution of the nucleic acid concentrations (ng/μL) obtained from (**A**) 943 samples showing a median value of 803.1 ng/μL and from (**B**) 738 samples after DNase treatment, showing a median value of 676.7 ng/μL. The concentrations were calculated from absorbance readings and ratios obtained using a Nanodrop2000. Most values fall within the first interquartile range (black vertical rectangle). White circle = Median values. The violin plot was obtained using Vioplot package. v 0.3.6. included in the R package v 3.6.1.

Virus detection from remote locations in the tropics must consider an optimized protocol for handling the samples under harsh environmental conditions during long hours of field work, long distance transport and long term storage for at least several weeks, until the samples reach the laboratory. The yield and quality of the nucleic acids obtained should be optimal for molecular diagnostics, especially for RNA viruses. In Fig. 1, we summarize the nucleic acid concentrations obtained using the protocol described here. The top youngest leaves of a cassava a plant produce a good quantity and quality of nucleic acids; they contain lower amounts of polysaccharides (starch), are easily identified in the plant and have been already proved to give a good representation of the viral infected status of the whole plant -even when these leaves do not usually show disease symptoms [5,8,18]. Drying out these leaves with silica gel right after they are collected, maintains the nucleic acids in good quality even after months in storage (Table 1), as has been shown in our previous works [2,9,11,13]. Transporting the samples in this way allows researchers to optimize the scouting by increasing the number of fields visited per day, while the samples are drying at room temperature.

**Table 1:** Average yield and quality of the nucleic acid extracts obtained in groups of samples that were stored for the same period in silica gel. (**A**) Samples stored for 6, 7 and 9 months. (**B**) Samples stored for 1.5 and 5 months and treated with DNase.

	Months stored in silica gel	Number of samples	Average concentration ng/µl	Average OD_260nm_/OD_280nm_	Average OD_260nm_/OD_230nm_
**A**	6	217	857.61 ± 255.29	2.09 ± 0.04	1.78 ± 0.18
	7	133	731.61 ± 232.97	2.10 ± 0.04	1.88 ± 0.17
	9	74	867.06 ± 414.09	2.05 ± 0.03	1.46 ± 0.20
**B**	1.5	60	645.85 ± 229.30	2.04 ± 0.07	1.89 ± 0.15
	5	60	644.92 ± 219.64	1.98 ± 0.06	1.87 ± 0.13

**Fig. 2:**
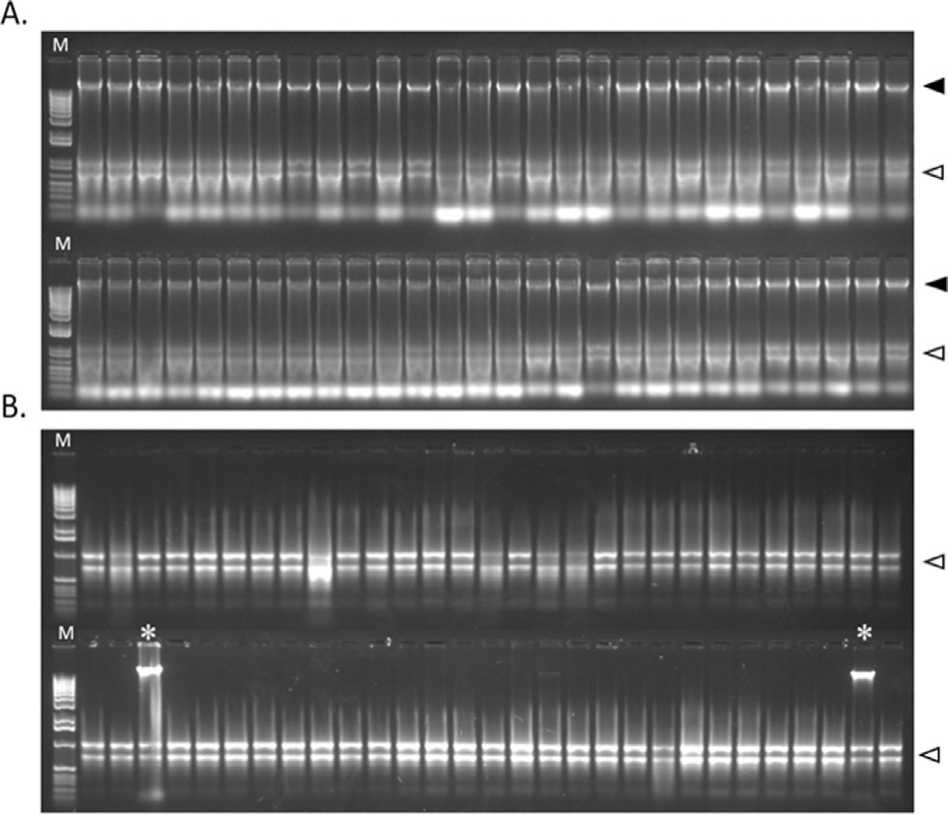
Agarose gel electrophoresis (1%) resolution of total nucleic acids extracted using the optimized protocol described in the text. (**A**) 4 μL of the extract were resolved at 120 V (90 mA) for 1 h in buffer TAE 1X. The black arrowheads point the location of the genomic DNA in the upper part of each gel and the white arrowheads indicate the location of the major ribosomal RNA bands. (**B**), Extracts after treatment with DNase. In few cases (asterisks) the treatment is not complete and needs to be repeated. M: 1 kb Plus DNA ladder.

We use Cetyltrimethylammonium-bromide (CTAB), a cationic detergent that under conditions of high ion concentrations, forms complexes with proteins and polysaccharides without precipitating nucleic acids. This characteristic is especially important when dealing with plant samples rich in polysaccharide and phenolic compound such as cassava [[15], [16], [17],^19^]. The use of silica gel, reusable carbon steel balls and the adaptation of a paint mixer to grind larger batches of samples at once allows one person to isolate total nucleic acids using the CTAB protocol, from 48 to 96 samples per day, depending on the equipment available (e.g. microcentrifuges). saving time and costs (a paint mixer is on average 4 times less expensive than e.g. a TissueLyzer II). This was also achievable by additional modifications to the original protocol [7]; we now do not use liquid Nitrogen nor phenol and to grind the samples in batches we use microfuge tubes instead of mortars and pestles, further saving costs (Supplementary Table 1-Costs). Working with large numbers of samples can increase the risk of losses during transport, drying or processing of the samples; we quantified the number of samples that did not yield an acceptable quality of nucleic acids needed for further analyses. Overall, out of 943 samples processed we obtained 0.42 % of losses. While for 738 samples treated with DNase, 3.1% were not optimal anymore for further analysis. These results were confirmed by agarose gel electrophoresis (Fig. 2). Quality and purity of total nucleic acid isolated were evaluated using absorbance ratios OD260/280 and OD260/230, which were above 2.0 and 1.5, respectively. After DNase treatment these values were still above 2.0 (OD260/280) and 1.5 (OD260/230) (Table 1). Total average yields obtained were 2.11 μg for total nucleic acids and 1.75 μg for total RNA, per milligram of dried leaf tissue. These values are comparable to those obtained in similar crops [1,19]. See Supplementary Table 2.

To test how long-term storage in silica gel would affect the results of the initial virus diagnostics we used two set of samples, one coming from plants infected with SLCMV (a DNA virus) and other infected with CsCMV (an RNA virus). We obtained a 97.4% of detection efficiency for SLCMV and 93.2% for CsCMV, in samples stored for at least 2 months in silica gel (Table 2). These are high efficiency detection values, and they most likely reflect the higher instability of RNA versus DNA. The addition of two extra steps for RT-PCR (DNase treatment and cDNA synthesis), may also increase the risk of obtaining false negatives for RNA viruses.

**Table 2:** Results of DNA and RNA virus detection from virus infected samples stored in silica gel for at least 2 months.

Virus	Virus infected samples	Samples positive after silica and CTAB ext.	% PCR Positives
SLCMV (DNA virus)	115	112	97.4
CsCMV (RNA virus)	44	41	93.2

Under field conditions vegetative propagated plants such as cassava, can be infected by several viruses (and other pathogens) over subsequent growing seasons [3,10,13]. Reverse transcriptase (RT) enzymes are among the most costly reagents in molecular diagnostics, which means that detection of RNA viruses tend also to be more expensive. To lower the costs we synthesize cDNA using random hexamer primers [14] which allows the detection of several virus species and strains, or even several genome regions of one virus, using the same cDNA reaction, as shown in previous works [2,20].

In conclusion, we describe the optimized protocol used in our laboratory to collect and store leaf samples for total nucleic acid isolation and molecular diagnostics of viruses. The total amounts of nucleic acid obtained even after storing the samples for several months in silica gel, were in all cases above the amounts needed for several rounds of virus diagnostics (Table 1), as is also evidenced by the PCR and RT-PCR results. It is noteworthy that nucleic acids obtained using this protocol have also been successfully used in our laboratory for isolation of small interfering RNA for Illumina sequencing [2,15], Rolling Circle Amplification of circular DNA and sequencing of viruses using Nanopore technology [5,12]. As the protocols applied here can also detect virus infections in asymptomatic plants [5,13,18], we suggest they could be part of a gold standard kit for CsCMV and SLCMV detection, and potentially for other viruses infecting cassava .

## Appendix Supplementary materials

Image, application 1

Image, application 2
