# Supplementary material for: An optimized nucleic acid isolation protocol for virus diagnostics in cassava (Manihot esculenta Crantz.)
Source: MethodsX. 2021 Aug 21;8:101496. doi: 10.1016/j.mex.2021.101496 (PMC8563463; doi:10.1016/j.mex.2021.101496)
Supplement: Supplementary file 1 [file mmc1.pdf]

Supplementary Table 1

A

Company	Name	Size	Price (USD)	Additional*	Cost per sample (USD)
Thermo fisher	PicoPure™ RNA Isolation Kit	40 samples	433.00	4.00	**10.92**
Qiagen	RNeasy Plant Mini Kit	50 samples	415.00	5.20	**8.40**
Qiagen	DNeasy Plant Mini Kit (50)	50 samples	392.00	5.20	**7.94**
OMEGA	E.Z.N.A.® DNA/RNA Isolation Kit	50 samples	359.30	5.20	**7.28**

*Mainly costs of plasticware

B

Reagent	Pack size	Price (USD)	Cost per 100 samples (USD)
NaCl 4 M	250 g	48.53	0.50
EDTA 0.5M	500 mL	52.75	0.50
Tris BASE	500 g	141.25	1.00
CTAB	100 g	35.00	60.00
PVP	1KG	261.00	0.40
β-mercaptoethanol	100 ml	37.42	0.50
BALINES DE 1/8	(PQ X 144 UN)	0.15	0.15
Chloroform	2.5L	48.61	1.94
Isopropanol	2.5L	32.00	1.28
Etanol	2.5L	30.76	1.24
Tubes 2 ml	500 Tubes	14.00	7.00
Tubes de 1.5 ml	500 Tubes	11.45	2.29
Tips with filter 1000 µL	960 tips in box	50.00	10.00
Tips with filter 200 µL	960 tips in box	26.00	6.00
			92.80
	**Cost per sample**		**0.928**
