# Supplementary material for: An optimized nucleic acid isolation protocol for virus diagnostics in cassava (Manihot esculenta Crantz.)
Source: MethodsX. 2021 Aug 21;8:101496. doi: 10.1016/j.mex.2021.101496 (PMC8563463; doi:10.1016/j.mex.2021.101496)
Supplement: Supplementary file 2 [file mmc2.pdf]

Supplementary Table 2

Young leaves plant tissue	Tissue	mg of tissue used	RNA yield (µg/g fresh tissue)	DNA yield (µg/mg fresh tissue)	RNA yield (µg/mg dry tissue)**	DNA yield (µg/mg dry tissue)	OD260nm/OD280nm	OD260nm/OD230nm	Reference
Sorghum bicolor	Lyophilized	~10 to 40				1.5±0.43	1.85±0.02	2.46±0.13	Xin & Chen, 2012
Gossypium hirsutum						1.0±0.11	1.85±0.01	2.25±0.08	
Zea mays						2.6±0.20	1.81±0.01	2.48±0.04	
Cynodondactylon						1±0.15	1.82±0.02	2.49±0.03	
Triticum astivum						2.5±0.42	1.85±0.02	2.46±0.04	
Populus deltoides						0.5±0.06	1.80±0.01	2.46±0.09	
Nicotiana tabacum						4.7±0.82	1.87±0.01	2.62±0.03	
Pinus eldarica						1.1±0.19	1.78±0.06	2.71±0.40	
Manihot esculenta	Fresh	1000	170 - 600		0.51 - 1.81		2.1	2.11	Behnam et al., 2019
*Corymbia citriodora subsp. variegata**	Fresh	1000		5		0.015	1.91	1.41	Healey et al, 2014
*Coffea brassii**		100		1.5 - 2.0		0.045 - 0.060	1.91	1.68	
Euphorbiaceae	Fresh	100	152–344		4.58 - 10.37		1.7 - 2.2	1.4 - 1.8	Xu et al. 2010
Manihot esculenta	**Dried**	**20**			**1.75 ±0.3**	**2.11 ±0.4**	**1.98 - 2.01**	**1.5 - 1.9**	**This study**

*The article does not mention the type of leaves used
± Standard deviation
**values considering 77.4% of water in fresh leaf tissue, as measured in our laboratory.
